# The Emerging Role of the RBM20 and PTBP1 Ribonucleoproteins in Heart Development and Cardiovascular Diseases

**DOI:** 10.3390/genes11040402

**Published:** 2020-04-08

**Authors:** Stefania Fochi, Pamela Lorenzi, Marilisa Galasso, Chiara Stefani, Elisabetta Trabetti, Donato Zipeto, Maria Grazia Romanelli

**Affiliations:** Department of Neurosciences, Biomedicine and Movement Sciences, Section of Biology and Genetics, University of Verona, 37134 Verona, Italy; stefania.fochi@univr.it (S.F.); pamela.lorenzi@univr.it (P.L.); marilisa.galasso@univr.it (M.G.); chiara.stefani@univr.it (C.S.); elisabetta.trabetti@univr.it (E.T.); donato.zipeto@univr.it (D.Z.)

**Keywords:** alternative splicing, ribonucleoproteins, RRM motif, PTBP1, RBM20, DCM, heart development, titin, RNA binding proteins, exon exclusion

## Abstract

Alternative splicing is a regulatory mechanism essential for cell differentiation and tissue organization. More than 90% of human genes are regulated by alternative splicing events, which participate in cell fate determination. The general mechanisms of splicing events are well known, whereas only recently have deep-sequencing, high throughput analyses and animal models provided novel information on the network of functionally coordinated, tissue-specific, alternatively spliced exons. Heart development and cardiac tissue differentiation require thoroughly regulated splicing events. The ribonucleoprotein RBM20 is a key regulator of the alternative splicing events required for functional and structural heart properties, such as the expression of TTN isoforms. Recently, the polypyrimidine tract-binding protein PTBP1 has been demonstrated to participate with RBM20 in regulating splicing events. In this review, we summarize the updated knowledge relative to RBM20 and PTBP1 structure and molecular function; their role in alternative splicing mechanisms involved in the heart development and function; RBM20 mutations associated with idiopathic dilated cardiovascular disease (DCM); and the consequences of RBM20-altered expression or dysfunction. Furthermore, we discuss the possible application of targeting RBM20 in new approaches in heart therapies.

## 1. Introduction

Splicing is a general mechanism that allows the removal of intron sequences from a precursor to mature mRNA. It is now accepted that, in higher eukaryotes, alternative splicing represents the major post-transcriptional mechanism that amplify the functional repertoire of expressed genes. In the human genome, ~95% of the genes containing introns are alternatively spliced [1]. The mechanism that allows alternative splicing has been intensively studied and several bioinformatics tools have been developed to predict the transcript variants of a specific gene [2,3]. However, not all the alternatively spliced transcripts can be translated into a protein, estimating that about 37% of human genes generate multiple protein isoforms [1]. While constitutive exon splicing requires ubiquitous splicing factors, which are components of the spliceosomal complex, recognizing highly conserved consensus sequences in introns, alternative splicing events are tightly regulated processes in cell responses and tissue differentiation [1,4,5,6]. It is not surprising that cells that do not undergo self-renewal, such as neurons and muscle cells, may be subjected to an intense alternative splicing activity during the life of the specific tissue [7,8,9,10]. 

Several regulatory factors can cooperate or compete in exon definition to allow the expression of tissues-specific transcript variants. Constitutive exon splicing derives from the spliceosomal recognition of conserved sequences at the 5′ splice site (ss), 3′ ss and branch site, whereas alternative exon recognition is regulated by *cis*-regulatory sequences in exon or intron components of the pre-mRNA, such as the exonic and intronic splicing enhancers, ESEs and ISEs, respectively, and the exonic and intronic splicing silencers, ESSs and ISSs. Enhancer elements may be recognized by *trans*-acting factors, such as SR proteins, a family of serine/arginine-rich family proteins, whereas silencing sequences may be generally recognized by the heterogeneous nuclear ribonucleoproteins (hnRNPs) [11,12]. SR proteins act generally as positive regulators, favoring exon inclusion, while hnRNPs most frequently act as negative regulators, leading to exon exclusion. A combinatorial binding of these factors in a position-dependent manner modulates the contribution of each factor, balancing their positive or negative effects on exon inclusion or exclusion [13]. Several databases and online tools concerning human alternative splicing and splice regulator factors, computationally generated or experimentally assessed, are available on websites, such as ASpedia, SpliceAid-F, MiasDB, SplicePort and HSF [14,15,16,17,18,19,20,21,22,23]. In recent years, intensive studies have contributed to identify several specific factors that participate in heart development as well as their involvement in heart diseases. Most of these factors, including transcription factors, constitutive proteins that accounts for cytoskeleton organization, electric impulse transmission, channel and cell-to-cell connection are regulated by alternative splicing [24,25]. RNA binding motif protein 20 (RBM20) has been identified as a key factor in driving the splicing events in transcripts that are selectively expressed in the heart [26,27,28,29]. Starting from genome-wide linkage analysis in two large families with autosomal dominant dilated cardiomyopathy (DCM), the identification of a mutation hotspot within RBM20 has directed the investigation on RBM20-regulated genes that may account for the development of cardiomyopathies [30]. Remarkably, RBM20 regulates alternative splicing of titin (*TTN*), one of the major disease-causing genes in cardiac muscle. Mutations or altered expression of RBM20 can lead to the shift in the expression pattern of titin transcript variants, which are associated with cardiac diseases, including DCM [31]. 

Based on recent studies, novel evidences have highlighted the role of the well-known splicing regulator polypyrimidine-tract binding protein 1 (PTBP1) in the regulation of alternatively spliced variants that are critical for the heart functionality [31,32]. In this review, we summarize the functional evidences of the role of RBM20 in the regulation of *TTN* and additional genes involved in heart function and cardiac diseases development. Furthermore, we review the current knowledge about the contribution of RBM20 and PTBP1 in heart alternative splicing events, their combinatory role in selecting specific exons and RBM20’s role in cardiovascular diseases. 

## 2. RBM20 Protein Structure

**Figure 1:**
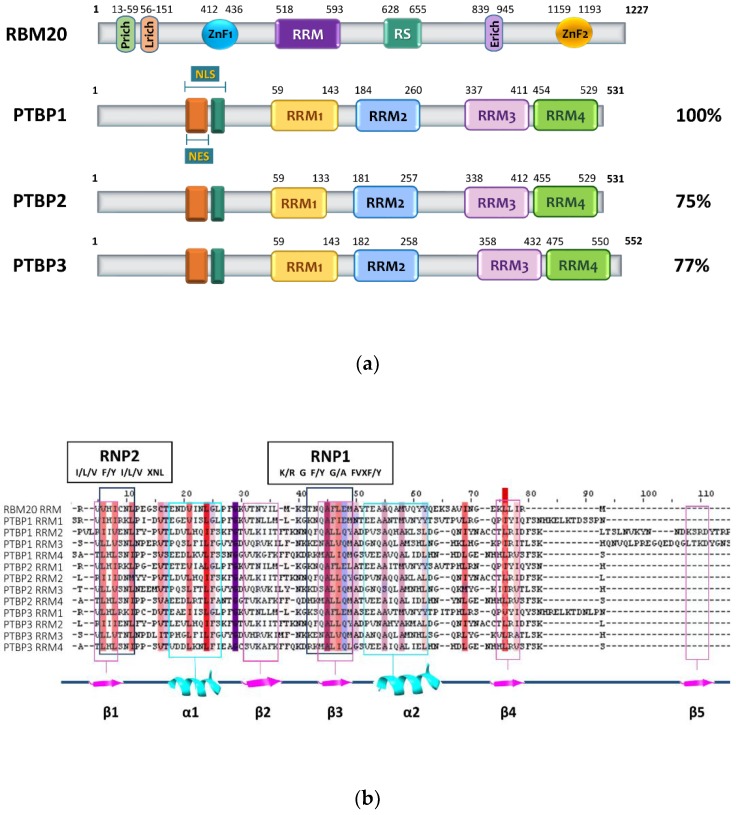
Schematic representation of the RBM20 and PTBP protein structures and multi-alignment of the RRM domains. (**a**) Numbers indicate the position of the amino acid residues relative to the protein domains. E-rich, glutamate-rich region; L-rich, leucine-rich region; P-rich, proline-rich region. RS, arginine/serine-rich region; ZnF1-2, zinc finger domains; NLS, nuclear localization signal; NES, nuclear export signal; RRM1 to 4, RNA-recognition motif domains. Percentage of homology of PTBP proteins is indicated relative to PTBP1. (**b**) Structure-based sequence alignment of the PTBP and RBM20 RRM domains. The alignment was performed by Clustal Omega analysis and edited using Jalview software [37]. Secondary structure elements predicted by the JPRED tool are indicated below the alignment. The RNA-binding domain cores, RNP1and RNP2, are indicated.

The RBM20 gene, located on chromosome 10 (10q25.2), encodes for a protein of 1227 amino acids and contains conserved functional domains: a leucine (L)-rich region at the N-terminus, two zinc finger (ZnF) domains (ZnF1 and ZnF2), an RNA recognition motif (RRM), an arginine–serine (RS) domain and a glutamate E-rich region between the RS domain and the ZnF2 domain at the C-terminal (Figure 1) [33,34,35,36]. We have demonstrated that RBM20 requires both the RRM and the RS-rich region to localize into the nucleus [34].

More recently, phosphorylation of the arginine–serine–arginine–serine–proline (RSRSP) stretch, within the RS domain, as well as their conservation, have been shown to be critical for RBM20 nuclear localization [35]. High-throughput sequencing and proteomics analyses indicate that RBM20 binds at multiple UCUU sites present at the 3′ and 5′ splice sites and it may interact with U1 and U2 small nuclear ribonucleic particles (snRNPs) and U2-related proteins, including U2AF65 and U2AF35 [38]. In the nuclei of mouse atrial myocyte HL-1 cells, RBM20 has been demonstrated to partially colocalize with PTBP1 and U2AF65 [33].

RBM20 is one of the few heart-specific splicing factors that has been demonstrated to regulate alternative splicing events of selected genes implicated in sarcomere assembly, ion transport and diastolic function [33]. Different types of alternative splicing events, including exon repression, mutually exclusive exon selection, exon inclusion, intron retention and exon shuffling are regulated by RBM20 [33,38,39]. The essential structural domains required for splicing activities are not fully identified, although RBM20 mutations in the RSRSP stretch and E-rich region have been demonstrated to affect exon splicing regulation [33,40]. Mutations at residues R634W and S635A of the RS-rich domain impair RBM20 nuclear localization, resulting in defective splicing regulation [33,35]. 

## 3. PTBP Proteins’ Structure and Function

The polypyrimidine tract-binding proteins (PTBPs) are ribonucleoproteins characterized by their ability to bind UC-rich regions within introns flanking regulated exons [41]. PTBP1, also known as hnRNP1 (heterogeneous nuclear ribonuclear protein I), was the first identified protein of the PTBP paralogs group, based on its property to bind to polypyrimidine sequences in precursor mRNAs [42,43,44,45]. PTBP1, widely expressed in tissues, is a shuttling protein between the nucleus and the cytoplasm that accumulates in the perinucleolar compartment (PNC) of the cells. [42,46]. PTBP1 is one of the most studied repressors of alternative splicing events. Beside its role in splicing processes, PTBP1 participates in several steps of RNA metabolism, including stability, polyadenylation, transport and cap-independent translation driven by internal ribosomal entry sites (IRESs) [41,47,48]. Tissue-specific PTBP1 roles are demonstrated in different tissues, including cardiomyocytes differentiation [49,50], neuronal development [51] and B lymphocytes selection in germinal center [52]. Furthermore, PTBP1 regulates microRNAs that repress neuronal-specific genes in non-neuronal cells. Depletion of PTBP1 in fibroblasts has been shown to induce fibroblast conversion into neurons by reprogramming the splicing events [53]. PTBP1 may be overexpressed in tumors, participating in proliferation control and migration of the cancer cells [54,55]. 

Differently from PTBP1, which is widely expressed in tissues and neuronal progenitor cells, the PTBP2 homolog, also known as nPTB, is mainly expressed in neurons and testis [56,57]. In neuronal differentiation, PTBP1 and PTBP2 undergo a programmed switch in their expression levels driving neuronal maturation [58,59,60,61]. When PTBP1 is highly expressed in the cell, it downregulates PTBP2 through an alternative splicing event that leads to nonsense-mediated decay (NMD) and PTBP2 mRNA degradation [61,62]. PTBP1 and PTBP2 share 74% sequence identity and a similar domain organization, represented by an N-terminal region containing a nuclear localization sequence (NLS), a nuclear export sequence (NES) [43,63,64] and four RNA recognition motifs (RRM1-4). RRM1 and RRM4 domains are folded in the canonical βαββαβ RRM structure, whereas in PTBP1 the RRM2 and RRM3 are extended by an additional fifth β-strand [65,66] (Figure 1). PTBP1 and PTBP2 bind UC-rich motifs, similarly to RBM20, although several RBM20 RRM residues differ from PTBP1 RRMs (Figure 1b). The repressive splicing activity of PTBP1 may be the result of different mechanisms that include blocking of spliceosome components, such as U2AF65 [67,68], hiding of the splice sites through the formation of oligomeric complexes [69] or by pre-mRNA modeling to favor the formation of loops that include regulated exons [70,71,72,73,74]. The binding to pyrimidine-rich motifs (e.g., UCUU and CUCUCU) mediated by the β sheets of PTBP1 RRM2 and 3, allows the identification of regulated exons [70,71,72].

PTBP1 and PTBP2 functions are modulated by interaction or competition with additional factors that may act as co-factors or competitors of PTBP1, such as Nova-1, Nova-2 [75], Raver1 [76,77], Raver2 [78,79] and MRG15 [80]. The interaction of PTB with Raver1 has been demonstrated to play a role in tissue splicing events, enhancing the smooth muscle-specific alternative splicing of alpha-tropomyosin (TM) exon 3 [76].

PTBP3, also known as ROD1 (Regulator Of Differentiation 1), has been discovered in hematopoietic cells [81]. PTBP3 binds preferentially to stretch of poly(G) and poly(U) sequences and is a regulator of EPO-dependent erythropoiesis. This PTBP paralog is highly expressed in hematopoietic cells and plays a role in the regulation of cell differentiation by repressing tissue-specific exons. It has been demonstrated that PTBP3 may promote exon 6 skipping in a Fas (Apo-1/CD-95) transcript with a comparable efficiency to PTBP1 and PTBP2 [82,83]. Recently, PTBP3 have been demonstrated to promote breast cancer epithelial–mesenchymal transition (EMT), promoting the expression of zinc finger transcription factor ZEB1, an EMT inducer, by binding to ZEB1 3′UTR and preventing its degradation [84]. PTBP3 may also play a pro-oncogenic role in gastric cancer cell cycle and growth, regulating the alternative splicing of transcription isomers of the Caveolin1 (CAV1) gene [85]. Furthermore, PTBP3 overexpression in human colorectal cancer has been demonstrated to enhance the expression of a cancer progression factor, the hypoxia-inducible transcription factors 1α (HIF-1α), by directly binding to the HIF-1α 5’UTR mRNA [86]. In pancreatic cancer cells, PTBP3 may promote tumor growth and resistance to chemotherapy by enhancing the mRNA stability of the autophagy-related gene ATG12 [87].

## 4. Regulation of Alternative Splicing Events in Heart by RBM20 and PTB

### 4.1. RBM20 Regulated Cardiac Pre-mRNAs

RBM20 exon targets have been initially identified by RNA-seq analyses in a *Rbm20-/-* rat’s heart and in human heart tissues derived from subjects carrying RBM20 mutations. These experimental approaches revealed a set of 31 genes regulated by RBM20 [33]. Photoactivable ribonucleoside-enhanced crosslinking-immunoprecipitation (PAR-CLIP) and high-throughput sequencing of immunoprecipitated RNA (HITS-CLIP) analyses, demonstrated a direct binding of RBM20 to stretches of UCUU pyrimidine in 18 genes differently regulated in cardiomyocytes derived from a *Rbm20-/-* rat heart compared to the wild type (wt) [38]. These wide analyses identified eight human and rat orthologous genes as RBM20 targets, most of them directly involved in heart function. These genes include the sarcomeric protein titin (*Tnt*), calcium/calmodulin dependent protein kinase II δ (*CaMKII-δ*), calcium voltage-gated channel Subunit α1 C calcium protein (*Cacna1c*), LIM domain binding 3 protein (*Ldb3*), LIM domain-only protein 7 (*Lmo7*), PDZ and LIM domain 5 protein (*Pdlim3*), reticulon 4 protein (*Rtn4*) and ryanodine receptor 2 (*Ryr2*).

Since the discovery of the first set of RBM20 target genes by RNA-seq, further studies have contributed to deepen the understanding of heart regulation mechanisms of transcript variants expression, extending the list of validated human or rat genes regulated by RBM20 (Table 1).

*TTN* is the most relevant gene regulated by RBM20 and truncating variants in titin are major determinants of heart disease, accounting for ~30% of DCM [97]. Titin is an essential sarcomeric component, spanning from the Z-line to M-line and being responsible for the passive elasticity of cardiac muscle [98]. The 363 exons of the *TTN* gene encode for the largest mammal protein, which is organized in modular domains consisting of ~300 immunoglobulin-like (Ig) and fibronectin-type III (FnIII) domains, an elastic proline (P), glutamate (E), valine (V) and lysine (K) (PEVK)-rich region in the I-band and a titin kinase (TK) domain located at the C-terminal [99,100]. The TTN pre-mRNA undergoes numerous alternative splicing events producing several titin isoforms that are tissue-specific and developmentally regulated. The best characterized isoforms are the cardiac and skeletal muscle isoforms named N2A, N2B and N2BA, which differ in the sequence and extension of the I-band domain [101]. N2B and N2BA are the two major adult cardiac titin isoforms that contribute to diastolic passive stiffness in the myocardium [101,102,103,104]. In the course of cardiac development, the frequency of TTN exon skipping shows a gradual increase, in favor of the shorter isoforms [105].

**Figure 2:**
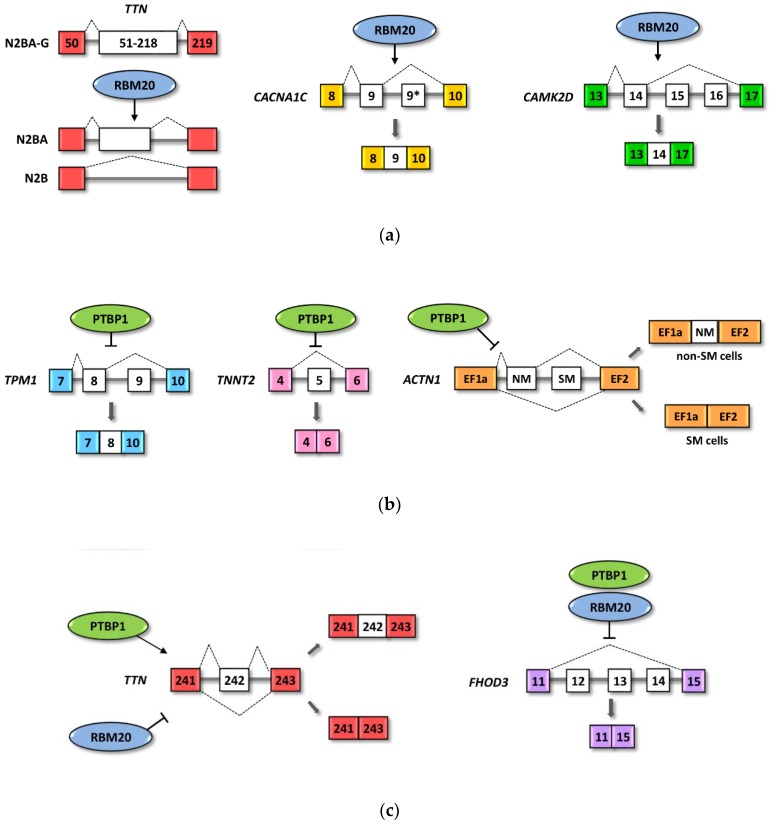
Schematic representation of examples of pre-mRNAs regulated by RBM20 and PTBP1. Colored exons represent constitutively spliced exons, while white exons represent alternative exons. (**a**) TTN (titin), CACNA1C (Calcium Voltage-Gated Channel Subunit Alpha1 C) and CAMK2D (Calcium/Calmodulin Dependent Protein Kinase II Delta) are examples of pre-mRNAs regulated by RBM20. (**b**) TPM1 (α-tropomyosin), TNNT2 (Troponin T2, Cardiac Type) and ACTN1 (α-actinin) are examples of pre-mRNAs regulated by PTBP1. (**c**) TTN (titin) and FHOD3 (Formin Homology 2 Domain Containing 3) are examples of pre-mRNAs regulated by both PTBP1 and RBM20.

The shift in protein expression from the long elastic TTN isoform N2A to the short, stiff isoforms N2B and N2BA, is coordinately regulated by alternative splicing during fetal to adult cardiac development. N2B is the smallest of the three isoforms, with a reduced number of Ig-like domains. In the absence of cardiac diseases N2B isoform is more expressed than N2BA [106]. The altered ratio between the titin isoforms is associated with cardiac diseases, including DCM [107]. Several studies demonstrate that in the absence of RBM20, the largest titin isoform, N2BA, is prevalently expressed during heart developmental phases, including the adult heart [33,88,108,109], while RBM20 overexpression is associated with an increased amount of the shorter, stiffer N2B isoform [33,39]. All these studies suggest that RBM20 acts as a repressor of exon inclusion in TTN isoforms participating to the switching in the TTN isoforms ratio N2BA:N2B during heart development and adult heart functionality (Figure 2a).

Insulin, the thyroid hormone T3 and Ang II have been demonstrated to regulate the alternatively spliced titin isoforms, involving the PI3K/AKT signaling pathway. The PI3K/AKT/mTor kinase axis has been shown to regulate titin isoform transition through increasing RBM20 expression [110,111,112]. Moreover, it has been demonstrated that the transcription factor ELK1 binds the RBM20 promoter and support a model in which Ang II may trigger ELK1 phosphorylation through the activation of MAPK signaling, promoting RBM20 expression [113].

A recent study proposes a new splicing-dependent mechanism regulated by RBM20 through the formation of circular RNA (circRNA) [114]. CircRNAs have been classified as noncoding RNA molecules, although recent studies demonstrate their ability to code for proteins [115]. CircRNAs are produced by the classical spliceosome machinery that covalently binds the 5′ and 3′ ends of an exon forming a stable single-strand RNA molecule that lack poly(A) tails [116]. It is thought that circRNAs are co-generated with mRNAs and their formation regulates gene expression competing with mRNA transcription to decrease the availability of linear mRNAs [117]. Khan et al. [114], exploring circRNA expression in the cardiac tissue from patients with hypertrophic cardiomyopathy (HCM) and DCM, identified a set of circRNAs produced by TTN. They demonstrated that RBM20 is required to produce circRNAs from the TTN I-band, a region extensively regulated by alternative splicing. 

Beside the RBM20-dependent alternative splicing of titin, the mechanism by which RBM20 regulates the expression of cardiac-specific genes has been deeply investigated in a limited number of studies. The functional role of RBM20 has been recently analyzed in the expression of three genes known to be alternatively spliced in the heart: CACNA1C encoding voltage-dependent L-calcium channel subunit alpha-1C (CaV1.2); CAMK2D encoding calcium/calmodulin-dependent protein kinase type II δ (CamkIIδ) and RYR2 encoding ryanodine receptor type 2 (RyR2). 

The CaV1.2 protein is a component of the pore unit of the cardiac L-type voltage-gated Ca2+ channels. In heart cells, influx of Ca2+ via CaV1.2 channels mediates excitation–contraction coupling, controls the action potential duration and regulates gene expression [118]. Dysregulation of the CaV1.2 activity, subcellular localization or surface density in cardiomyocytes can result in cardiac arrhythmias and heart failure [119,120]. CaV1.2 isoforms are differentially expressed during heart development and possess different electrophysiological properties. RBM20 overexpression in neonatal rat cardiomyocytes promotes the inclusion of exon 9* in CACNA1C mRNA and its skipping when RBM20 is silenced [89]. Functional data indicated that RBM20 expression affects the level of L-type voltage-gated Ca2+ currents regulating the inclusion of exon 9*. RBM20 directly binds to consensus sequences in the flanking intron region of exon 9* and, when overexpressed, reduces CaV1.2 membrane surface expressions [89].

CamkIIδ is a multifunctional Ser/Thr protein kinase, which regulates excitation–contraction coupling. In the adult heart, CamkIIδ is expressed in multiple isoforms and altered expression of CAMK2D variants are associated with cardiac hypertrophy and heart failure [121]. The CamkIIδ functions differ based on its nuclear or cytoplasmic localization. In the cytoplasm, CamkIIδ phosphorylates RyR2 and phospholamban, whereas in the nucleus it associates with histone deacetylases (HDACs) to regulate transcription factor expression [121]. The inclusion of exon 14 leads to nuclear translocation of the CamkIIδ isoforms. Cardiomyocytes from *Rbm20-/-* mice show aberrant splicing events that lead to a switch of the smallest CamkIIδ isoforms toward the biggest isoform, CaMKII-δA, which includes exon 15 and 16, but not exon 14, thus altering their relative intracellular distributions [122]. Similarly to CAMK2D, RBM20 regulates the expression of a nuclear Ryr2 isoform containing an exon of 24 pb that determines its intranuclear distribution [123]. Neonatal rat Rbm20-/- cardiomyocytes show, in fact, a shift in the expression of the RYR2 isoform containing the 24 bp exon [122]. An interesting study by Bertero et al. [88] proposes a mechanism by which RBM20 generates a splicing factory, selecting cardiac-specific trans-interacting domains (TIDs) located on different chromosomes. This splicing factory regulates the alternative splicing of genes associated with the TTN locus. They demonstrated that TTN, CACNA1C and CAMK2D are present in the RBM20-dependent trans-acting chromatin domain and are coordinately regulated in their alternative splicing events. They propose that the TTN pre-mRNA, due to the concentration of RBM20 target motifs, may act as a scaffold for RBM20 foci of the TTN locus, in which selected genes, such as CACNA1C and CAMK2D, are co-coordinately regulated. In human embryonic stem-cell-derived cardiomyocytes, the knockout of RBM20 affects the choice of alternative exons for both the TTN-associated genes CACNA1C and CAMK2D. RBM20 KO results in exon 9* inclusion in CACNA1C mRNA and an increased ratio of the CaMKIIδA isoform over CaMKIIδB, which presents the inclusion of exons 14 (Figure 2a) [88]. All these studies add new evidence in support of the critical role of RBM20 in the regulation of genes required for heart functionality.

### 4.2. Role of RBM20 in Heart Diseases

Direct involvement of RBM20 in heart disease is proved by two orders of evidence. First, animal models deficient in the expression of *Rbm20* develop CDM [33,122]; secondly, RBM20 mutations have been identified in 2–3% of familial and sporadic cases of DCM [28,30,124,125,126,127]. The mutations identified in DCM cases are principally heterozygous missense mutations and most of them lie within the RS-domain where a hotspot of mutations is located in the RSRSP stretch [28]. Mutations in the TTN gene are the main cause of familial DCM [100]. Most of these mutations result in the repression of the PEVK exons of titin, leading to the formation of the giant N2BA-G isoform enriched in DCM. The stiffness of the myocardial wall during ventricular filling is controlled by the passive tension of the titin protein that arise from the proper isoform ratio N2A:N2B. *Rbm20-/-* rats, showing an increased ratio of N2BA:N2B, develop DCM [33], whereas the induced expression of RBM20 in *Rbm20-/-* rats decreases the N2BA:N2B ratio, thus suggesting that the reduction of RBM20 expression levels may lead to DCM in humans. Moreover, a reduced expression of RBM20 derived by mutations or experimental knockout models, leads to ventricular dilatation [30,33,124]. In mouse models it has been demonstrated that heterozygous RBM20 mutations have intermediate effects on titin length, passive force and slack sarcomere length, indicating that the amount of the wt RBM20 can be crucial for the structural and functional properties of heart tissue [128]. The possibility of rescuing the altered ratio N2BA:N2B, enhancing the expression of the shortest stiffer N2B isoform targeting RBM20, opens a new prospective in developing potential therapeutic strategies.

A recent study has investigated the initial molecular aberration in RBM20-mediated DCM, examining the effect of RBM20 point mutations using human pluripotent stem cell-derived cardiomyocytes (hPSC-CMs) [129]. The authors induced pluripotent stem cell (iPSC), derived from somatic cells of DCM patients harboring the RBM20 R636S missense mutation, to differentiate into cardiomyocytes (CMs) (hiPSC-CMs). Transcriptome profiling analyses in the hiPSC-CMs confirms that several RBM20-dependent splice variants were altered, including TTN, LDB3, CAMK2D and CACNA1C. RBM20 hiPSC-CMs present defects in the calcium-handling machinery. In addition, the structural assessment of RBM20 hiPSC-CMs revealed an increase in the sarcomeric length and a decrease in the sarcomeric width. An additional cell model has been derived by iPSC-CMs from patients harboring the S635A RBM20 missense mutation [130]. This RBM20-hiPSC-CMs present an irregular distribution of the sarcomeric protein α-actinin and defective calcium handling. Interestingly, the authors observed a reduction of TTN exon skipping in the PEVK region, resulting in a reduced expression of the N2B isoform, in line with the results obtained by Bertero et al. [88], demonstrating that RBM20 is required for the exclusion of PEVK exons. The iPSCs harboring RBM20 human mutations offer a great opportunity for modeling heart disease in vitro. Interesting contributions on the possibility of therapeutic application targeting RBM20 are also derived from studies on heterozygous RBM20 KO mice. In these animal models the balance between beneficial and disadvantageous effects derived by altered titin isoform expression, favors the positive effect of more compliant titin in the reduction of diastolic chamber stiffness [128,131]. RBM20’s role in titin splicing regulation has also been analyzed in relation to diastolic dysfunction, observing that reduced RBM20 activity improves diastolic dysfunction [132]. To discover a drug that may improve cardiac elastic titin isoform expression, more than 34,0000 small molecules have been screened in splice reporter assays. These analyses identified cardenolides as inhibitors of RBM20-mediated titin splicing, opening the possibility to treat diastolic heart failure by modulating titin splicing through drugs targeting RBM20 [40]. The mechanisms that regulate the expression of RBM20 remain unclarified and the identification of factors that may cooperate with RBM20 in splicing regulation has just started. Further studies are needed in order to select RBM20 as an exclusive target in controlling the heart splicing network. 

### 4.3. PTBP1 Regulated Heart Pre-mRNA

The expression level of PTBP1 is associated with neuronal development and muscle differentiation [56,133]. Wide analyses of PTB targets in HeLa cells indicate that PTBP1 represses many neuronal and striated muscle-specific exons in genes encoding cytoskeletal and signaling proteins, highlighting its role in the fine regulation of protein isoform expression required for cell differentiation [134,135,136]. PTBP1 generally determines the repression of selected exons in alternative splice variants (Figure 2b). In mice, PTBP1 expression has been demonstrated to be gradually reduced during heart development and cardiomyocyte differentiation [50]. 

Some aspects of the mechanism that regulates PTB expression have been partially elucidated. In differentiating neurons, a switch between the expression of PTBP1 and the neuronal paralog PTBP2 has been demonstrated to be driven by the neuronal micro-RNA miR-124, which downregulates PTBP1 expression, leading to a series of neuronal specific alternative splicing events [61,137]. In C2C12 cells, a mouse myoblast cell line, the protein RBM4, has been demonstrated to promote the skipping of PTBP1 exon 11 as well as the skipping of neuronal PTBP2 exon 10, leading to nonsense-mediated mRNA decay (NMD) and consequentially reducing PTBP protein levels [138]. In addition, during differentiation of C2C12 myoblasts, the increased expression of miR-133 downregulates the expression of PTBP2 by targeting its 3’UTR and thus altering the splicing of several mRNAs involved in muscle maturation [62]. PTBP1 has been demonstrated to be highly expressed in the murine myocardium during embryonic cardiac development and progressively repressed after birth [50]. In this animal model the overexpression of PTBP1 in postnatal cardiomyocytes induces an increase in pro-apoptotic protein expression without altering the abundance of their mRNA [50]. By means of a bicistronic plasmid, the authors provided evidence that PTBP1 overexpression in neonatal cardiomyocytes enhances the IRES-dependent translation, suggesting that PTBP1 may regulate the expression of pro-apoptotic proteins, such as caspases, at the translational level. In an interesting study, using rat primary cardiomyocytes and caspase-deficient mice, the same research group has demonstrated a novel signaling network, involving histone deacetylases (HDACs) and caspase activity, by which PTBP1 levels are reduced during neuronal developments [93]. They propose that HDACs expression levels may regulate the caspase activity, leading to PTBP1 cleavage and degradation by proteasomes during cardiac development. Furthermore, they demonstrate that during cardiomyocytes differentiation, the levels of PTBP1 regulate exon expression of the tropomyosin 1 and 2 (*TPM1* and *TPM2*) transcript variants. The reduced PTBP1 levels may thus influence the use of alternatively regulated exons in heart transcript isoforms. Tropomyosin is one of the structural proteins of the thin sarcomere filaments and it is responsible for mediating the Ca2+ control of contraction and relaxation [139]. Mutually exclusive exons of *Tpm1* and *Tpm2* have been previously demonstrated to be regulated by PTBP1 [91,96,134,140,141]. Overexpression of PTBP1 in neonatal cardiomyocytes induces the exclusion of exon 9 in *TPM1* (Figure 2b) and exon 7 in *TPM2*. Reduction of PTBP1 during early postnatal heart development directly correlates with the expression of *TPM1* exon 9 and *TPM2* exon 7 in myocardium isoforms. Furthermore, variation of PTBP1 expression during heart development influences the myocyte enhancer factors-2 (MEF2), determining the inclusion of exon β in Mef2a and Mef2b, which represent the most abundant transcript variants in the adult heart [93]. 

PTBP1 regulates the alternative splicing of two additional pre-mRNAs relevant for cardiomyocyte function, Troponin-T (*TNNT2*) and α-actinin (*ACTN1*) [90,94]. TNNT2 mediates muscle contraction in response to calcium ion dynamics. Mutations in the *TNNT2* gene have been associated with multiple types of cardiomyopathy [142]. The cardiac troponin T (cTNT) pre-mRNA contains a single alternative exon, exon 5, which introduces an additional 10 amino acids in the protein sequence, conferring a higher sensibility to calcium [143]. Exon 5 is predominantly included in mRNAs produced in the embryonic heart, while it is excluded in the adult heart [144]. Intronic Muscle Specific Elements (MSEs) required for exon inclusion in embryonic skeletal muscle culture are located upstream and downstream of exon 5. Charlet-B et al. [94] demonstrated that PTBP1 represses the exon 5 inclusion of TNNT2 in an MSE-dependent manner, in primary embryonic skeletal muscle cultures (Figure 2b). In cardiomyocytes, PTBP1 and muscleblind-like (MBNL) proteins have been proposed to antagonize the effect of CUG-binding protein (CUG-BP) and ETR-3-like factor (CELF) RNA binding proteins, regulating cTNT exon 5 skipping [145]. 

The actin-crosslinking protein α-actinin 1, encoded by the ACTN1 gene, is a homodimeric molecule that contains three functional domains: an N-terminal region containing two actin-binding motifs, a central dimer-forming sequence with four spectrin-like domains and a C-terminal region with two EF hand motifs. Biochemical data have provided evidence that binding of ACTN1 to actin is controlled by the first EF hand motif [146]. Different tissues express at least two isoforms of ACTN1 that differ in the expression of tissue-specific, alternative splicing of mutually exclusive exons. The non-muscle exon (NM) codes for 27 amino acids that form the C-terminus of the first EF hand, while smooth muscle cells contain a specific exon (SM) that codes for 22 different amino acids. In vitro experiments by depletion and rescue of PTBP1 expression showed that PTBP1 regulates the alternative splicing of the α-actinin mutually exclusive SM and NM exons [90,91,147]. PTBP1 induces the exclusion of the α-actinin SM exon in the majority of the variants expressed in non-smooth muscle cells (non-SM cells), leading to the inclusion of the alternative upstream NM exon, whereas in smooth muscle cells PTBP1 determines the exclusion of both SM and NM exons (Figure 2b). In a mouse model of cardiac hypertrophy, the level of PTBP1 expression, as well as epithelial splicing regulatory proteins (ESRP1) and SF2/ASF splicing factors, have been observed to be significantly altered [148], suggesting that the imbalance amount of these splicing factors may lead to a disequilibrium in the expression of tissue-specific regulated genes.

### 4.4. RBM20 and PTBP1 Combinatorial Effects on Alternative Splicing 

The balance between splicing activation and repression mediated by splicing factors, as well as the combinatorial effect of RNA-binding competition or promotion are expected to play a crucial role in directing regulated alternative splicing events in tissues differentiation. A study that investigated the molecular bases of TTN exon exclusion regulated by RBM20, using a splicing reporter and in vitro binding assay, demonstrated that the PTBP1 isoform PTB4 regulates titin splicing [31]. PTB4 may counteract the RBM20 splicing repressor activity, binding the same consensus motif on the 5′ splice site (5′SS), located downstream of the TTN 242 alternative exon (Figure 2c). Binding of both PTB4 and RBM20 to the downstream intron may differentially interfere with U1 snRNP, favoring the inclusion of the alternative exon [31]. 

We recently investigated the involvement of RBM20 and PTBP1 in the regulation of the alternative splicing of the formin homology 2 domain containing 3 (FHOD3) protein, an RNA-seq-predicted RBM20 target [33]. FHOD3 is a sarcomeric protein expressed in the cardiac tissue that regulates actin dynamics [149,150]. Mutations in the human FHOD3 gene have been associated with hypertrophic cardiomyopathy (HCM) and DCM [151,152]. We found that both RBM20 and PTBP1 influence the balance of the FHOD3 splicing pattern, promoting the skipping of exons 12, 13 and 14 (Figure 2c). Furthermore, we observed a positive correlation between FHOD3 exon 12 skipping and the overexpression of RBM20 or PTBP1. We hypothesize that they both participate in the splice site recognition by competing with the snRNP spliceosomal components that determine the exon inclusion/exclusion outcome [32].

**Figure 3:**
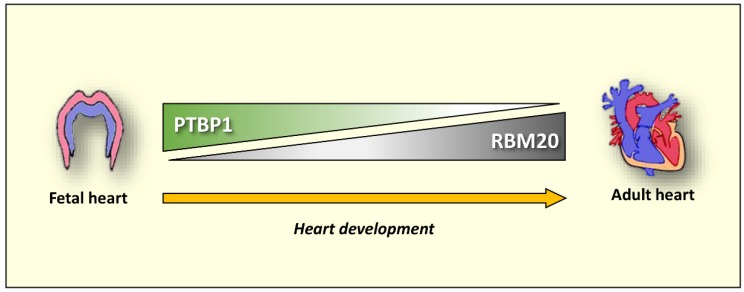
Schematic representation of RBM20 and PTBP1 temporal expression during heart development.

It is interesting to note that RBM20 and PTBP1 expression are inversely correlated during heart development. While RBM20 expression increases during heart development, PTBP1 levels are reduced [50,93,153]. We may expect that a regulatory mechanism participates in the dynamic of the temporal switch of RBM20 and PTBP1 expression during heart differentiation, inhibiting the expression of the ubiquitous PTBP1 in favor of a more selective and tissue-specific RBM20 splicing factor (Figure 3). Further studies can address the knowledge gap on how these factors may cooperate to control heart-specific gene expression during differentiation and heart disease development. Moreover, it will be interesting to explore the combined role of the PTBP1 co-factors, such as Raver1 and Raver2, as well as additional splicing factors, such as CELF, MBNL, Rbfox and SF3B1 in regulating the splicing events in cardiac variants. 

### 4.5. RBM20 and RBM24 Cooperation in Alternative Splicing 

Other than RBM20 and PTBP1, additional splicing factors are known to be specifically involved in the regulation of splicing events required for heart muscular functionality, which include (MBNL1), CUG-binding protein 1 (CUGBP1), RNA-binding protein fox homolog 1 (RBFOX1) and RNA-binding motif protein 24 (RBM24) [29]. Mutations affecting their expression and functionality have been identified in HCM, DCM and heart failure [154,155,156]. An interesting functional cooperation between RBM20 and RBM24 have been recently demonstrated. RBM20 together with RBM24 promotes the inclusion of exon 11 of Enigma homolog (ENH) protein, resulting in the shorter ENH splice variants. These shorter isoforms, lacking LIM domains, are suggested to prevent HCM [157]. RBM24 is required for normal heart development, and when knocked out, causes early mice death [51,158,159]. In animal models, RBM24 is required for sarcomere assembly and heart contractility [160]. Recently, Liu et al. [161] showed that RBM24 deletion in a mouse model resulted in the missplicing of several genes coding for sarcomere structure proteins, such as *Tpm2, Ttn, Nebl, Fhod3, Enah* and *Ablim1*. These authors provide evidence that RBM24 binds *Ttn* pre-mRNA, altering the expression of the cardiac isoforms. In their model the knockout of RBM24 in the postnatal heart leads to rapidly progressive DCM, heart failure and postnatal lethality. A commentary on this study evidenced that RBM24 mutations in human cardiomyopathy patients might be a rare event due to the absence of RBM24 mutations associated with human disease [162]. In rat cardiomyocytes, RBM20 and RBM24 have been also demonstrated to cooperate to regulate the splicing events in the scaffold proteins expressed by the Eng gene. Both proteins promote the expression of the Eng isoforms lacking the LIM domain, which prevented cardiomyocyte hypertrophy in a mouse model [157,163]. A similar cooperation between RBM24 and PTBP1 has been proposed for exon inclusion in splicing variants, suggesting that the balance between tissue-specific splicing factors, such as RBM24, and the widespread expressed factors, such as PTBP1, plays a critical role in controlling splicing events [158]. 

## 5. Conclusions and Future Perspectives 

Global transcriptomic studies have evidenced the role of RBM20 in the regulation of expressed splicing isoforms in heart tissues and functional studies are actually deciphering the mechanism of specific RBM20 target exon regulation. Since the mechanisms of alternative splicing regulation are now more extensively investigated, the tissue-specific splicing patterns are beginning to be unraveled. However, additional studies are required to define the complexity of exon regulation in heart development. The identification of key splicing factors, such as RBM20 and PTBP1 and their function in the heart, is now contributing to insights into the mechanisms that lead to heart disease. Advances in high-throughput sequence technologies and computational algorithms for data analysis have expanded the effectiveness in investigating the complex splicing network active in heart development and differentiation. However, functional analyses of the expressed isoforms, as well as their role and combinatorial action on alternative splicing, are essential to demonstrate the consequences of potential splice-disrupting mutations. Recent studies on reprogrammed iPS cells are contributing to identifying the temporal steps that drive, by alternative splicing, the tissue differentiation and represent a promising model to recapitulate tissue-specific isoform expression. Besides, improving the detection of splicing events and the identification of the RNA consensus sequences recognized by splicing factors, may provide the bases for the development of RNA-targeted therapies suitable for heart diseases, such as splice-switching antisense oligonucleotides or short interfering RNAs. It will be of interest to investigate the role of splicing events regulating noncoding RNAs, as well as to develop 3D heart organoids to decipher the splicing code of heart development and cardiomyopathies based on alterations in titin function. The next challenge in developing heart disease therapies will be extending functional studies to clarify the effect of human mutations in the alternative isoforms regulated by the splicing machinery in the heart.

## Figures and Tables

**Table 1:** Representative heart genes regulated by RBM20 and PTBP1.

Gene Symbol^a^	Gene Name	Regulated Exons	Species	References
**RBM20-regulated genes**				
*CACNA1C*	Calcium Voltage-Gated Channel Subunit α1 C	9	Human ESC *RBM20* KO CMs	[88]
		9*	Rat cardiomyocytes	[89]
*CAMK2D*	Calcium/calmodulin dependent protein kinase II delta	14	Human cardiac *RBM20* (S635A) tissue, *Rbm20*-/- rats, Human ESC *RBM20* KO CMs	[33]
*CAMKIIG*	Calcium/calmodulin dependent protein kinase II gamma	12–15	Human cardiac RBM20 (S635A) tissue, *Rbm20*-/- rats	[33]
*FHOD3*	Formin homology 2 domain containing 3	12–14	HeLa cells	[32]
*LDB3*	LIM domain binding 3	4, 5/6	Human cardiac *RBM20* (S635A) tissue, *Rbm20*-/- rats	[33]
*LMO7*	LIM domain only protein 7	9, 10	*Rbm20*-/- rats	[38]
*MLIP*	Muscular-enriched A type laminin-interacting protein	9, 10	*Rbm20*-/- rats	[38]
*PDLIM3*	PDZ and LIM domain 3	4–6	*Rbm20*-/- rats	[38]
*RTN4*	Reticulon 4	3, 4	*Rbm20*-/- rats	[38]
*RYR2*	Ryanodine receptor 2	24 bp exon	*Rbm20*-/- rats	[38]
*SH3KBP1*	SH3 domain containing kinase binding protein 1	6–7	Human cardiac *RBM20* (S635A) tissue, *Rbm20*-/- rats	[33]
*SORBS1*	Sorbin and SH3 domain containing 1	2	Human cardiac *RBM20* (S635A) tissue, *Rbm20*-/- rats	[33]
*TRDN*	Triadin	8	Human cardiac RBM20 (S635A) tissue, *Rbm20*-/- rats	[33]
*TTN*	Titin	PEVK exons	Human cardiac *RBM20* (S635A) tissue, *Rbm20*-/- rats, Human ESC *RBM20* KO CMs	[33,88]
**PTBP1-regulated genes**				
*ACTN1*	α-actinin	NM/SM	Non-smooth muscle cells, PAC1 smooth muscle cells	[90,91]
*CACNA1C*	Calcium Voltage-Gated Channel Subunit α1 C	8/8a	Neuro-2a cells	[92]
*FHOD3*	Formin homology 2 domain containing 3	12–14	HeLa cells	[32]
*MEF2*	Myocyte Enhancer Factor 2	3	Rat cardiomyocytes	[93]
*TNNT2*	Troponin T type 2	5	Primary embryonic skeletal muscle cultures	[94]
*TPM1*	Tropomyosin 1	3	PAC1 smooth muscle cells	[91,95]
		9	Rat cardiomyocytes	[93]
*TPM2*	Tropomyosin 2	7	HeLa cells, rat cardiomyocytes	[93,96]
*TTN*	Titin	242	HEK293 cells	[31]

^a^ Genes underlined are commonly regulated by RBM20 and PTBP1. CM: cardiomyocytes; ESC: embryonic stem cell.

## References

[1] Baralle F.E., Giudice J. (2017). Alternative splicing as a regulator of development and tissue identity. Nat. Rev. Mol. Cell Biol..

[2] Ding L., Rath E., Bai Y. (2017). Comparison of Alternative Splicing Junction Detection Tools Using RNASeq Data. Curr. Genom..

[3] Ohno K., Takeda J.I., Masuda A. (2018). Rules and tools to predict the splicing effects of exonic and intronic mutations. Wiley Interdiscip. Rev. RNA.

[4] Licatalosi D.D., Darnell R.B. (2010). RNA processing and its regulation: Global insights into biological networks. Nat. Rev. Genet..

[5] Kalsotra A., Cooper T.A. (2011). Functional consequences of developmentally regulated alternative splicing. Nat. Rev. Genet..

[6] Ellis J.D., Barrios-Rodiles M., Çolak R., Irimia M., Kim T.H., Calarco J.A., Wang X., Pan Q., O’Hanlon D., Kim P.M. (2012). Tissue-Specific Alternative Splicing Remodels Protein-Protein Interaction Networks. Mol. Cell.

[7] Barbosa-Morais N.L., Irimia M., Pan Q., Xiong H.Y., Gueroussov S., Lee L.J., Slobodeniuc V., Kutter C., Watt S., Çolak R. (2012). The evolutionary landscape of alternative splicing in vertebrate species. Science.

[8] Traunmüller L., Gomez A.M., Nguyen T.M., Scheiffele P. (2016). Control of neuronal synapse specification by a highly dedicated alternative splicing program. Science.

[9] Hinkle E.R., Wiedner H.J., Black A.J., Giudice J. (2019). RNA processing in skeletal muscle biology and disease. Transcription.

[10] Nikonova E., Kao S.-Y., Spletter M.L. (2020). Contributions of alternative splicing to muscle type development and function. Semin. Cell Dev. Biol..

[11] Wang Y., Liu J., Huang B., Xu Y.-M., Li J., Huang L.-F., Lin J., Zhang J., Min Q.-H., Yang W.-M. (2015). Mechanism of alternative splicing and its regulation. Biomed. Rep..

[12] Shenasa H., Hertel K.J. (2019). Combinatorial regulation of alternative splicing. Biochim. Biophys. Acta Gene Regul. Mech..

[13] Ule J., Blencowe B.J. (2019). Alternative Splicing Regulatory Networks: Functions, Mechanisms, and Evolution. Mol. Cell.

[14] Aspedia http://combio.snu.ac.kr/aspedia/.

[15] Hyung D., Kim J., Cho S.Y., Park C. (2018). ASpedia: A comprehensive encyclopedia of human alternative splicing. Nucleic Acids Res..

[16] SpliceAdF http://srv00.recas.ba.infn.it/SpliceAidF/.

[17] Giulietti M., Piva F., D’Antonio M., De Meo P.D.O., Paoletti D., Castrignanò T., D’Erchia A.M., Picardi E., Zambelli F., Principato G. (2013). SpliceAid-F: A database of human splicing factors and their RNA-binding sites. Nucleic Acids Res..

[18] MiasDB http://47.88.84.236/Miasdb/.

[19] Xing Y., Zhao X., Yu T., Liang D., Li J., Wei G., Liu G., Cui X., Zhao H., Cai L. (2016). MiasDB: A database of molecular interactions associated with alternative splicing of human pre-mRNAs. PLoS ONE.

[20] SplicePort https://spliceport.cbcb.umd.edu/.

[21] Dogan R.I., Getoor L., Wilbur W.J., Mount S.M. (2007). SplicePort-An interactive splice-site analysis tool. Nucleic Acids Res..

[22] HSF http://www.umd.be/HSF/.

[23] Desmet F.O., Hamroun D., Lalande M., Collod-Bëroud G., Claustres M., Béroud C. (2009). Human Splicing Finder: An online bioinformatics tool to predict splicing signals. Nucleic Acids Res..

[24] Wang H., Chen Y., Li X., Chen G., Zhong L., Chen G., Liao Y., Liao W., Bin J. (2016). Genome-wide analysis of alternative splicing during human heart development. Sci. Rep..

[25] Beqqali A. (2018). Alternative splicing in cardiomyopathy. Biophys. Rev..

[26] Ma J., Lu L., Guo W., Ren J., Yang J. (2016). Emerging Role for RBM20 and its Splicing Substrates in Cardiac Function and Heart Failure. Curr. Pharm. Des..

[27] Weeland C.J., Van den Hoogenhof M.M., Beqqali A., Creemers E.E. (2015). Insights into alternative splicing of sarcomeric genes in the heart. J. Mol. Cell. Cardiol..

[28] Watanabe T., Kimura A., Kuroyanagi H. (2018). Alternative splicing regulator RBM20 and cardiomyopathy. Front. Mol. Biosci..

[29] Rexiati M., Sun M., Guo W. (2018). Muscle-specific Mis-splicing and heart disease exemplified by RBM20. Genes.

[30] Brauch K.M., Karst M.L., Herron K.J., De Andrade M., Pellikka P.A., Rodeheffer R.J., Michels V.V., Olson T.M. (2009). Mutations in Ribonucleic Acid Binding Protein Gene Cause Familial Dilated Cardiomyopathy. J. Am. Coll. Cardiol..

[31] Dauksaite V., Gotthardt M. (2018). Molecular basis of titin exon exclusion by RBM20 and the novel titin splice regulator PTB4. Nucleic Acids Res..

[32] Lorenzi P., Sangalli A., Fochi S., Dal Molin A., Malerba G., Zipeto D., Romanelli M.G. (2019). RNA-binding proteins RBM20 and PTBP1 regulate the alternative splicing of FHOD3. Int. J. Biochem. Cell Biol..

[33] Guo W., Schafer S., Greaser M.L., Radke M.H., Liss M., Govindarajan T., Maatz H., Schulz H., Li S., Parrish A.M. (2012). RBM20, a gene for hereditary cardiomyopathy, regulates titin splicing. Nat. Med..

[34] Filippello A., Lorenzi P., Bergamo E., Romanelli M.G. (2013). Identification of nuclear retention domains in the RBM20 protein. FEBS Lett..

[35] Murayama R., Kimura-Asami M., Togo-Ohno M., Yamasaki-Kato Y., Naruse T.K., Yamamoto T., Hayashi T., Ai T., Spoonamore K.G., Kovacs R.J. (2018). Phosphorylation of the RSRSP stretch is critical for splicing regulation by RNA-Binding Motif Protein 20 (RBM20) through nuclear localization. Sci. Rep..

[36] Zahr H.C., Jaalouk D.E. (2018). Exploring the crosstalk between LMNA and splicing machinery gene mutations in Dilated Cardiomyopathy. Front. Genet..

[37] Waterhouse A.M., Procter J., Martin D., Clamp M., Barton G. (2009). Jalview Version 2-A multiple sequence alignment editor and analysis workbench. Bioinformatics.

[38] Maatz H., Jens M., Liss M., Schafer S., Heinig M., Kirchner M., Adami E., Rintisch C., Dauksaite V., Radke M.H. (2014). RNA-binding protein RBM20 represses splicing to orchestrate cardiac pre-mRNA processing. J. Clin. Investig..

[39] Li S., Guo W., Dewey C.N., Greaser M.L. (2013). Rbm20 regulates titin alternative splicing as a splicing repressor. Nucleic Acids Res..

[40] Liss M., Radke M.H., Eckhard J., Neuenschwander M., Dauksaite V., Von Kries J.P., Gotthardt M. (2018). Drug discovery with an RBM20 dependent titin splice reporter identifies cardenolides as lead structures to improve cardiac filling. PLoS ONE.

[41] Romanelli M.G., Diani E., Lievens P.M.J. (2013). New insights into functional roles of the polypyrimidine tract-binding protein. Int. J. Mol. Sci..

[42] Ghetti A., Piñol-Roma S., Michael W.M., Morandi C., Dreyfuss G. (1992). hnRNP I, the polypyrimidine tract-binding protein: Distinct nuclear localization and association with hnRNAs. Nucleic Acids Res..

[43] Romanelli M.G., Weighardt F., Biamonti G., Riva S., Morandi C. (1997). Sequence determinants for hnRNP I protein nuclear localization. Exp. Cell Res..

[44] Romanelli M.G., Lorenzi P., Morandi C. (2000). Organization of the human gene encoding heterogeneous nuclear ribonucleoprotein type I (hnRNP I) and characterization of hnRNP I related pseudogene. Gene.

[45] Oberstrass F.C., Auwetor S.D., Erat M., Hargous Y., Henning A., Wenter P., Reymond L., Amir-Ahmady B., Pitsch S., Black D.L. (2005). Structural biology—Structure of PTB bound to RNA: Specific binding and implications for splicing regulation. Science.

[46] Romanelli M.G., Faggioli L., Lorenzi P., Morandi C. (2001). Cloning and functional characterization of the human heterogeneous nuclear ribonucleoprotein type I promoter. Biochim. Biophys. Acta.

[47] Wollerton M.C., Gooding C., Wagner E.J., Garcia-Blanco M.A., Smith C.W.J. (2004). Autoregulation of Polypyrimidine Tract Binding Protein by Alternative Splicing Leading to Nonsense-Mediated Decay. Mol. Cell.

[48] Sawicka K., Bushell M., Spriggs K.A., Willis A.E. (2008). Polypyrimidine-tract-binding protein: A multifunctional RNA-binding protein. Biochem. Soc. Trans..

[49] Kalsotra A., Xiao X., Ward A.J., Castle J.C., Johnson J.M., Burge C.B., Cooper T.A. (2008). A postnatal switch of CELF and MBNL proteins reprograms alternative splicing in the developing heart. Proc. Natl. Acad. Sci. USA.

[50] Zhang J., Bahi N., Llovera M., Comella J.X., Sanchis D. (2009). Polypyrimidine tract binding proteins (PTB) regulate the expression of apoptotic genes and susceptibility to caspase-dependent apoptosis in differentiating cardiomyocytes. Cell Death Differ..

[51] Zhang X., Chen M.H., Wu X., Kodani A., Fan J., Doan R., Ozawa M., Ma J., Yoshida N., Reiter J.F. (2016). Cell-Type-Specific Alternative Splicing Governs Cell Fate in the Developing Cerebral Cortex. Cell.

[52] Monzón-Casanova E., Screen M., Díaz-Muñoz M.D., Coulson R.M.R., Bell S.E., Lamers G., Solimena M., Smith C.W.J., Turner M. (2018). The RNA-binding protein PTBP1 is necessary for B cell selection in germinal centers article. Nat. Immunol..

[53] Xue Y., Ouyang K., Huang J., Zhou Y., Ouyang H., Li H., Wang G., Wu Q., Wei C., Bi Y. (2013). Direct conversion of fibroblasts to neurons by reprogramming PTB-regulated MicroRNA circuits. Cell.

[54] Takahashi H., Nishimura J., Kagawa Y., Kano Y., Takahashi Y., Wu X., Hiraki M., Hamabe A., Konno M., Haraguchi N. (2015). Significance of polypyrimidine tract-binding protein 1 expression in colorectal cancer. Mol. Cancer Ther..

[55] Xie R., Chen X., Chen Z., Huang M., Dong W., Gu P., Zhang J., Zhou Q., Dong W., Han J. (2019). Polypyrimidine tract binding protein 1 promotes lymphatic metastasis and proliferation of bladder cancer via alternative splicing of MEIS2 and PKM. Cancer Lett..

[56] Keppetipola N., Sharma S., Li Q., Black D.L. (2012). Neuronal regulation of pre-mRNA splicing by polypyrimidine tract binding proteins, PTBP1 and PTBP2. Crit. Rev. Biochem. Mol. Biol..

[57] Romanelli M.G., Lorenzi P., Morandi C. (2005). Identification and analysis of the human neural polypyrimidine tract binding protein (nPTB) gene promoter region. Gene.

[58] Raj B., Blencowe B.J. (2015). Alternative Splicing in the Mammalian Nervous System: Recent Insights into Mechanisms and Functional Roles. Neuron.

[59] Ling J.P., Chhabra R., Merran J.D., Schaughency P.M., Wheelan S.J., Corden J.L., Wong P.C. (2016). PTBP1 and PTBP2 Repress Nonconserved Cryptic Exons. Cell Rep..

[60] Li Q., Zheng S., Han A., Lin C.H., Stoilov P., Fu X.D., Black D.L. (2014). The splicing regulator PTBP2 controls a program of embryonic splicing required for neuronal maturation. Elife.

[61] Makeyev E.V., Zhang J., Carrasco M.A., Maniatis T. (2007). The MicroRNA miR-124 Promotes Neuronal Differentiation by Triggering Brain-Specific Alternative Pre-mRNA Splicing. Mol. Cell.

[62] Boutz P.L., Stoilov P., Li Q., Lin C.H., Chawla G., Ostrow K., Shiue L., Ares M., Black D.L. (2007). A post-transcriptional regulatory switch in polypyrimidine tract-binding proteins reprograms alternative splicing in developing neurons. Genes Dev..

[63] Romanelli M.G., Morandi C. (2002). Importin α binds to an unusual bipartite nuclear localization signal in the heterogeneous ribonucleoprotein type I. Eur. J. Biochem..

[64] Li B., Benedict Yen T.S. (2002). Characterization of the nuclear export signal of polypyrimidine tract-binding protein. J. Biol. Chem..

[65] Conte M.R., Grüne T., Ghuman J., Kelly G., Ladas A., Matthews S., Curry S. (2000). Structure of tandem RNA recognition motifs from polypyrimidine tract binding protein reveals novel features of the RRM fold. EMBO J..

[66] Simpson P.J., Monie T.P., Szendröi A., Davydova N., Tyzack J.K., Conte M.R., Read C.M., Cary P.D., Svergun D.I., Konarev P.V. (2004). Structure and RNA interactions of the N-terminal RRM domains of PTB. Structure.

[67] Lin C.H., Patton J.G. (1995). Regulation of alternative 3′ splice site selection by constitutive splicing factors. RNA.

[68] Singh R., Valcárcel J., Green M.R. (1995). Distinct binding specificities and functions of higher eukaryotic polypyrimidine tract-binding proteins. Science.

[69] Wagner E.J., Garcia-Blanco M.A. (2001). Polypyrimidine tract binding protein antagonizes exon definition. Mol. Cell. Biol..

[70] Chou M.Y., Underwood J.G., Nikolic J., Luu M.H., Black D.L. (2000). Multisite RNA binding and release of polypyrimidine tract binding protein during the regulation of c-src neural-specific splicing. Mol. Cell.

[71] Izquierdo J.M., Majós N., Bonnal S., Martínez C., Castelo R., Guigó R., Bilbao D., Valcárcel J. (2005). Regulation of fas alternative splicing by antagonistic effects of TIA-1 and PTB on exon definition. Mol. Cell.

[72] Cherny D., Gooding C., Eperon G.E., Coelho M.B., Bagshaw C.R., Smith C.W.J., Eperon I.C. (2010). Stoichiometry of a regulatory splicing complex revealed by single-molecule analyses. EMBO J..

[73] Sharma S., Kohlstaedt L.A., Damianov A., Rio D.C., Black D.L. (2008). Polypyrimidine tract binding protein controls the transition from exon definition to an intron defined spliceosome. Nat. Struct. Mol. Biol..

[74] Sharma S., Maris C., Allain F.H.T., Black D.L. (2011). U1 snRNA Directly Interacts with Polypyrimidine Tract-Binding Protein during Splicing Repression. Mol. Cell.

[75] Polydorides A.D., Okano H.J., Yang Y.Y.L., Stefani G., Darnell R.B. (2000). A brain-enriched polypyrimidine tract-binding protein antagonizes the ability of Nova to regulate neuron-specific alternative splicing. Proc. Natl. Acad. Sci. USA.

[76] Gromak N., Rideau A., Southby J., Scadden A.D.J., Gooding C., Hüttelmaier S., Singer R.H., Smith C.W.J. (2003). The PTB interacting protein raver1 regulates alpha-tropomyosin alternative splicing. EMBO J..

[77] Romanelli M.G., Lorenzi P., Avesani F., Morandi C. (2007). Functional characterization of the ribonucleoprotein, PTB-binding 1/Raver1 promoter region. Gene.

[78] Kleinhenz B., Fabienke M., Swiniarski S., Wittenmayer N., Kirsch J., Jockusch B.M., Arnold H.H., Illenberger S. (2005). Raver2, a new member of the hnRNP family. FEBS Lett..

[79] Romanelli M.G., Lorenzi P., Diani E., Filippello A., Avesani F., Morandi C. (2012). Transcriptional regulation of the human raver2 ribonucleoprotein gene. Gene.

[80] Luco R.F., Pan Q., Tominaga K., Blencowe B.J., Pereira-Smith O.M., Misteli T. (2010). Regulation of alternative splicing by histone modifications. Science.

[81] Yamamoto H., Tsukahara K., Kanaoka Y., Jinno S., Okayama H. (1999). Isolation of a Mammalian Homologue of a Fission Yeast Differentiation Regulator. Mol. Cell. Biol..

[82] Robinson F., Jackson R.J., Smith C.W.J. (2008). Expression of Human nPTB Is Limited by Extreme Suboptimal Codon Content. PLoS ONE.

[83] Tan K.S., Inoue T., Kulkeaw K., Tanaka Y., Lai M.I., Sugiyama D. (2015). Localized SCF and IGF-1 secretion enhances erythropoiesis in the spleen of murine embryos. Biol. Open.

[84] Hou P., Li L., Chen F., Chen Y., Liu H., Li J., Bai J., Zheng J. (2018). PTBP3-Mediated regulation of zeb1 mRNA stability promotes epithelial–mesenchymal transition in breast cancer. Cancer Res..

[85] Liang X., Chen W., Shi H., Gu X., Li Y., Qi Y., Xu K., Zhao A., Liu J. (2018). PTBP3 contributes to the metastasis of gastric cancer by mediating CAV1 alternative splicing. Cell Death Dis..

[86] Hou P., Chen F., Yong H., Lin T., Li J., Pan Y., Jiang T., Li M., Chen Y., Song J. (2019). PTBP3 contributes to colorectal cancer growth and metastasis via translational activation of HIF-1α. J. Exp. Clin. Cancer Res..

[87] Ma J., Weng L., Jia Y., Liu B., Wu S., Xue L., Yin X., Mao A., Wang Z., Shang M. (2020). PTBP3 promotes malignancy and hypoxia-induced chemoresistance in pancreatic cancer cells by ATG12 up-regulation. J. Cell. Mol. Med..

[88] Bertero A., Fields P.A., Ramani V., Bonora G., Yardimci G.G., Reinecke H., Pabon L., Noble W.S., Shendure J., Murry C.E. (2019). Dynamics of genome reorganization during human cardiogenesis reveal an RBM20-dependent splicing factory. Nat. Commun..

[89] Morinaga A., Ito J., Niimi T., Maturana A.D. (2019). RBM20 regulates CaV1.2 surface expression by promoting exon 9* Inclusion of CACNA1C in neonatal rat cardiomyocytes. Int. J. Mol. Sci..

[90] Southby J., Gooding C., Smith C.W.J. (1999). Polypyrimidine Tract Binding Protein Functions as a Repressor To Regulate Alternative Splicing of α-Actinin Mutally Exclusive Exons. Mol. Cell. Biol..

[91] Wollerton M.C., Gooding C., Robinson F., Brown E.C., Jackson R.J., Smith C.W.J. (2001). Differential alternative splicing activity of isoforms of polypyrimidine tract binding protein (PTB). RNA.

[92] Tang Z.Z., Sharma S., Zheng S., Chawla G., Nikolic J., Black D.L. (2011). Regulation of the mutually exclusive exons 8a and 8 in the CaV1.2 calcium channel transcript by polypyrimidine tract-binding protein. J. Biol. Chem..

[93] Ye J., Llorian M., Cardona M., Rongvaux A., Moubarak R.S., Comella J.X., Bassel-Duby R., Flavell R.A., Olson E.N., Smith C.W.J. (2013). A pathway involving HDAC5, cFLIP and caspases regulates expression of the splicing regulator polypyrimidine tract binding protein in the heart. J. Cell Sci..

[94] Charlet-B N., Logan P., Singh G., Cooper T.A. (2002). Dynamic antagonism between ETR-3 and PTB regulates cell type-specific alternative splicing. Mol. Cell.

[95] Gooding C., Roberts G.C., Smith C.W.J. (1998). Role of an inhibitory pyrimidine element and polypyrimidine tract binding protein in repression of a regulated α-tropomyosin exon. RNA.

[96] Mulligan G.J., Guo W., Wormsley S., Helfman D.M. (1992). Polypyrimidine tract binding protein interacts with sequences involved in alternative splicing of beta-tropomyosin pre-mRNA. J. Biol. Chem..

[97] LeWinter M.M., Granzier H.L. (2013). Titin is a major human disease gene. Circulation.

[98] Hidalgo C., Granzier H. (2013). Tuning the molecular giant titin through phosphorylation: Role in health and disease. Trends Cardiovasc. Med..

[99] Labeit S., Kolmerer B. (1995). Titins: Giant proteins in charge of muscle ultrastructure and elasticity. Science.

[100] Linke W.A., Hamdani N. (2014). Gigantic business: Titin properties and function through thick and thin. Circ. Res..

[101] Freiburg A., Trombitas K., Hell W., Cazorla O., Fougerousse F., Centner T., Kolmerer B., Witt C., Beckmann J.S., Gregorio C.C. (2000). Series of exon-skipping events in the elastic spring region of titin as the structural basis for myofibrillar elastic diversity. Circ. Res..

[102] Granzier H.L., Labeit S. (2004). The Giant Protein Titin: A Major Player in Myocardial Mechanics, Signaling, and Disease. Circ. Res..

[103] Lewinter M.M. (2014). Acute pericarditis. N. Engl. J. Med..

[104] Guo W., Zhu C., Yin Z., Wang Q., Sun M., Cao H., Greaser M.L. (2018). Splicing factor RBM20 regulates transcriptional network of titin associated and calcium handling genes in the heart. Int. J. Biol. Sci..

[105] Opitz C.A., Linke W.A. (2005). Plasticity of Cardiac Titin/Connectin in Heart Development. J. Muscle Res. Cell Motil..

[106] Nagueh S.F., Shah G., Wu Y., Torre-Amione G., King N.M.P., Lahmers S., Witt C.C., Becker K., Labeit S., Granzier H.L. (2004). Altered titin expression, myocardial stiffness, and left ventricular function in patients with dilated cardiomyopathy. Circulation.

[107] Bozkurt B., Colvin M., Cook J., Cooper L.T., Deswal A., Fonarow G.C., Francis G.S., Lenihan D., Lewis E.F., McNamara D.M. (2016). Current Diagnostic and Treatment Strategies for Specific Dilated Cardiomyopathies: A Scientific Statement from the American Heart Association. Circulation.

[108] Guo W., Pleitner J.M., Saupe K.W., Greaser M.L. (2013). Pathophysiological defects and transcriptional profiling in the RBM20 -/- rat model. PLoS ONE.

[109] Li S., Guo W., Schmitt B.M., Greaser M.L. (2012). Comprehensive analysis of titin protein isoform and alternative splicing in normal and mutant rats. J. Cell. Biochem..

[110] Krüger M., Babicz K., Von Frieling-Salewsky M., Linke W.A. (2010). Insulin signaling regulates cardiac titin properties in heart development and diabetic cardiomyopathy. J. Mol. Cell. Cardiol..

[111] Zhu C., Yin Z., Ren J., McCormick R.J., Ford S.P., Guo W. (2015). RBM20 is an essential factor for thyroid hormone-regulated titin isoform transition. J. Mol. Cell Biol..

[112] Zhu C., Yin Z., Tan B., Guo W. (2017). Insulin regulates titin pre-mRNA splicing through the PI3K-Akt-mTOR kinase axis in a RBM20-dependent manner. Biochim. Biophys. Acta Mol. Basis Dis..

[113] Cai H., Zhu C., Chen Z., Maimaiti R., Sun M., McCormick R.J., Lan X., Chen H., Guo W. (2019). Angiotensin ii influences pre-mRNA splicing regulation by enhancing RBM20 transcription through activation of the MAPK/ELK1 signaling pathway. Int. J. Mol. Sci..

[114] Khan M.A.F., Reckman Y.J., Aufiero S., Van Den Hoogenhof M.M.G., Van Der Made I., Beqqali A., Koolbergen D.R., Rasmussen T.B., Van Der Velden J., Creemers E.E. (2016). RBM20 Regulates Circular RNA Production from the Titin Gene. Circ. Res..

[115] Pamudurti N.R., Bartok O., Jens M., Ashwal-Fluss R., Stottmeister C., Ruhe L., Hanan M., Wyler E., Perez-Hernandez D., Ramberger E. (2017). Translation of CircRNAs. Mol. Cell.

[116] Memczak S., Jens M., Elefsinioti A., Torti F., Krueger J., Rybak A., Maier L., Mackowiak S.D., Gregersen L.H., Munschauer M. (2013). Circular RNAs are a large class of animal RNAs with regulatory potency. Nature.

[117] Ashwal-Fluss R., Meyer M., Pamudurti N.R., Ivanov A., Bartok O., Hanan M., Evantal N., Memczak S., Rajewsky N., Kadener S. (2014). CircRNA Biogenesis competes with Pre-mRNA splicing. Mol. Cell.

[118] Catterall W.A. (2000). Structure and Regulation of Voltage-Gated Ca^2+^ Channels. Annu. Rev. Cell Dev. Biol..

[119] Yang L., Dai D.F., Yuan C., Westenbroek R.E., Yu H., West N., De La Iglesia H.O., Catterall W.A. (2016). Loss of β-adrenergic-stimulated phosphorylation of CaV1.2 channels on Ser1700 leads to heart failure. Proc. Natl. Acad. Sci. USA.

[120] Zhang Q., Chen J., Qin Y., Wang J., Zhou L. (2018). Mutations in voltage-gated L-type calcium channel: Implications in cardiac arrhythmia. Channels.

[121] Gray C.B.B., Heller Brown J. (2014). CaMKIIdelta subtypes: Localization and function. Front. Pharmacol..

[122] Van Den Hoogenhof M.M.G., Beqqali A., Amin A.S., Van Der Made I., Aufiero S., Khan M.A.F., Schumacher C.A., Jansweijer J.A., Van Spaendonck-Zwarts K.Y., Remme C.A. (2018). RBM20 mutations induce an arrhythmogenic dilated cardiomyopathy related to disturbed calcium handling. Circulation.

[123] George C.H., Rogers S.A., Bertrand B.M.A., Tunwell R.E.A., Thomas N.L., Steele D.S., Cox E.V., Pepper C., Hazeel C.J., Claycomb W.C. (2007). Alternative splicing of ryanodine receptors modulates cardiomyocyte Ca^2+^ signaling and susceptibility to apoptosis. Circ. Res..

[124] Li D., Morales A., Gonzalez-Quintana J., Norton N., Siegfried J.D., Hofmeyer M., Hershberger R.E. (2010). Identification of novel mutations in RBM20 in patients with dilated cardiomyopathy. Clin. Transl. Sci..

[125] Refaat M.M., Lubitz S.A., Makino S., Islam Z., Frangiskakis J.M., Mehdi H., Gutmann R., Zhang M.L., Bloom H.L., MacRae C.A. (2012). Genetic variation in the alternative splicing regulator RBM20 is associated with dilated cardiomyopathy. Hear. Rhythm.

[126] Kayvanpour E., Sedaghat-Hamedani F., Amr A., Lai A., Haas J., Holzer D.B., Frese K.S., Keller A., Jensen K., Katus H.A. (2017). Genotype-phenotype associations in dilated cardiomyopathy: Meta-analysis on more than 8000 individuals. Clin. Res. Cardiol..

[127] Beqqali A., Bollen I.A.E., Rasmussen T.B., Van den Hoogenhof M.M., Van Deutekom H.W.M., Schafer S., Haas J., Meder B., Sørensen K.E., Van Oort R.J. (2016). A mutation in the glutamate-rich region of RNA-binding motif protein 20 causes dilated cardiomyopathy through missplicing of titin and impaired Frank-Starling mechanism. Cardiovasc. Res..

[128] Methawasin M., Hutchinson K.R., Lee E.J., Smith J.E., Saripalli C., Hidalgo C.G., Ottenheijm C.A.C., Granzier H. (2014). Experimentally increasing titin compliance in a novel mouse model attenuates the frank-starling mechanism but has a beneficial effect on diastole. Circulation.

[129] Wyles S.P., Li X., Hrstka S.C., Reyes S., Oommen S., Beraldi R., Edwards J., Terzic A., Olson T.M., Nelson T.J. (2016). Modeling structural and functional deficiencies of RBM20 familial dilated cardiomyopathy using human induced pluripotent stem cells. Hum. Mol. Genet..

[130] Streckfuss-Bömeke K., Tiburcy M., Fomin A., Luo X., Li W., Fischer C., Özcelik C., Perrot A., Sossalla S., Haas J. (2017). Severe DCM phenotype of patient harboring RBM20 mutation S635A can be modeled by patient-specific induced pluripotent stem cell-derived cardiomyocytes. J. Mol. Cell. Cardiol..

[131] Bull M., Methawasin M., Strom J., Nair P., Hutchinson K., Granzier H. (2016). Alternative splicing of titin restores diastolic function in an HFpEF-like genetic murine model (Ttn ΔIAjxn). Circ. Res..

[132] Hinze F., Dieterich C., Radke M.H., Granzier H., Gotthardt M. (2016). Reducing RBM20 activity improves diastolic dysfunction and cardiac atrophy. J. Mol. Med..

[133] Llorian M., Gooding C., Bellora N., Hallegger M., Buckroyd A., Wang X., Rajgor D., Kayikci M., Feltham J., Ule J. (2016). The alternative splicing program of differentiated smooth muscle cells involves concerted non-productive splicing of post-transcriptional regulators. Nucleic Acids Res..

[134] Llorian M., Schwartz S., Clark T.A., Hollander D., Tan L.Y., Spellman R., Gordon A., Schweitzer A.C., De La Grange P., Ast G. (2010). Position-dependent alternative splicing activity revealed by global profiling of alternative splicing events regulated by PTB. Nat. Struct. Mol. Biol..

[135] Spellman R., Llorian M., Smith C.W.J. (2007). Crossregulation and Functional Redundancy between the Splicing Regulator PTB and Its Paralogs nPTB and ROD1. Mol. Cell.

[136] Xue Y., Zhou Y., Wu T., Zhu T., Ji X., Kwon Y.S., Zhang C., Yeo G., Black D.L., Sun H. (2009). Genome-wide Analysis of PTB-RNA Interactions Reveals a Strategy Used by the General Splicing Repressor to Modulate Exon Inclusion or Skipping. Mol. Cell.

[137] Boutz P.L., Chawla G., Stoilov P., Black D.L. (2007). MicroRNAs regulate the expression of the alternative splicing factor nPTB during muscle development. Genes Dev..

[138] Lin J.C., Tarn W.Y. (2011). RBM4 down-regulates PTB and antagonizes its activity in muscle cell-specific alternative splicing. J. Cell Biol..

[139] Dube D.K., McLean M.D., Dube S., Poiesz B.J. (2014). Translational control of tropomyosin expression in vertebrate hearts. Anat. Rec..

[140] Patton J.G., Mayer S.A., Tempst P., Nadal-Ginard B. (1991). Characterization and molecular cloning of polypyrimidine tract-binding protein: A component of a complex necessary for pre-mRNA splicing. Genes Dev..

[141] Llorian M., Smith C.W.J. (2011). Decoding muscle alternative splicing. Curr. Opin. Genet. Dev..

[142] McConnell M., Tal Grinspan L., Williams M.R., Lynn M.L., Schwartz B.A., Fass O.Z., Schwartz S.D., Tardiff J.C. (2017). Clinically Divergent Mutation Effects on the Structure and Function of the Human Cardiac Tropomyosin Overlap. Biochemistry.

[143] Godt R.E., Fogaça R.T., Silva I.K., Nosek T.M. (1993). Contraction of developing avian heart muscle. Comp. Biochem. Physiol. Comp. Physiol..

[144] Cooper T.A., Ordahl C.P. (1985). A single cardiac troponin T gene generates embryonic and adult isoforms via developmentally regulated alternate splicing. J. Biol. Chem..

[145] Ladd A.N., Stenberg M.G., Swanson M.S., Cooper T.A. (2005). Dynamic balance between activation and repression regulates pre-mRNA alternative splicing during heart development. Dev. Dyn..

[146] Waites G.T., Graham I.R., Jackson P., Millake D.B., Patel B., Blanchard A.D., Weller P.A., Eperon I.C., Critchley D.R. (1992). Mutually exclusive splicing of calcium-binding domain exons in chick alpha-actinin. J. Biol. Chem..

[147] Matlin A.J., Southby J., Gooding C., Smith C.W.J. (2007). Repression of α-actinin SM exon splicing by assisted binding of PTB to the polypyrimidine tract. RNA.

[148] Kim T., Kim J.O., Oh J.G., Hong S.E., Kim D.H. (2014). Pressure-overload cardiac hypertrophy is associated with distinct alternative splicing due to altered expression of splicing factors. Mol. Cells.

[149] Taniguchi K., Takeya R., Suetsugu S., Kan-o M., Narusawa M., Shiose A., Tominaga R., Sumimoto H. (2009). Mammalian formin Fhod3 regulates actin assembly and sarcomere organization in striated muscles. J. Biol. Chem..

[150] Ushijima T., Fujimoto N., Matsuyama S., Kan-O M., Kiyonari H., Shioi G., Kage Y., Yamasaki S., Takeya R., Sumimoto H. (2018). The actin-organizing formin protein Fhod3 is required for postnatal development and functional maintenance of the adult heart in mice. J. Biol. Chem..

[151] Wooten E.C., Hebl V.B., Wolf M.J., Greytak S.R., Orr N.M., Draper I., Calvino J.E., Kapur N.K., Maron M.S., Kullo I.J. (2013). Formin homology 2 domain containing 3 variants associated with hypertrophic cardiomyopathy. Circ. Cardiovasc. Genet..

[152] Arimura T., Takeya R., Ishikawa T., Yamano T., Matsuo A., Tatsumi T., Nomura T., Sumimoto H., Kimura A. (2013). Dilated cardiomyopathy-associated FHOD3 variant impairs the ability to induce activation of transcription factor serum response factor. Circ. J..

[153] Beraldi R., Li X., Fernandez A.M., Reyes S., Secreto F., Terzic A., Olson T.M., Nelson T.J. (2014). Rbm20-deficient cardiogenesis reveals early disruption of RNA processing and sarcomere remodeling establishing a developmental etiology for dilated cardiomyopathy. Hum. Mol. Genet..

[154] Lara-Pezzi E., Gómez-Salinero J., Gatto A., García-Pavía P. (2013). The alternative heart: Impact of alternative splicing in heart disease. J. Cardiovasc. Transl. Res..

[155] Kong S.W., Hu Y.W., Ho J.W.K., Ikeda S., Polster S., John R., Hall J.L., Bisping E., Pieske B., Dos Remedios C.G. (2010). Heart Failure-Associated Changes in RNA Splicing of Sarcomere Genes. Circ. Cardiovasc. Genet..

[156] Giudice J., Xia Z., Wang E.T., Scavuzzo M.A., Ward A.J., Kalsotra A., Wang W., Wehrens X.H.T., Burge C.B., Li W. (2014). Alternative splicing regulates vesicular trafficking genes in cardiomyocytes during postnatal heart development. Nat. Commun..

[157] Ito J., Iijima M., Yoshimoto N., Niimi T., Kuroda S., Maturana A.D. (2016). RBM20 and RBM24 cooperatively promote the expression of short enh splice variants. FEBS Lett..

[158] Yang J., Hung L.H., Licht T., Kostin S., Looso M., Khrameeva E., Bindereif A., Schneider A., Braun T. (2014). RBM24 Is a major regulator of muscle-specific alternative splicing. Dev. Cell.

[159] Zhang M., Zhang Y., Xu E., Mohibi S., De Anda D.M., Jiang Y., Zhang J., Chen X. (2018). Rbm24, a target of p53, is necessary for proper expression of p53 and heart development. Cell Death Differ..

[160] Poon K.L., Tan K.T., Wei Y.Y., Ng C.P., Colman A., Korzh V., Xu X.Q. (2012). RNA-binding protein RBM24 is required for sarcomere assembly and heart contractility. Cardiovasc. Res..

[161] Liu J., Kong X., Zhang M., Yang X., Xu X. (2019). RNA binding protein 24 deletion disrupts global alternative splicing and causes dilated cardiomyopathy. Protein Cell.

[162] Gaertner A., Brodehl A., Milting H. (2019). Screening for mutations in human cardiomyopathy- is RBM24 a new but rare disease gene?. Protein Cell.

[163] Maturana A.D., Nakagawa N., Yoshimoto N., Tatematsu K., Hoshijima M., Tanizawa K., Kuroda S. (2011). LIM domains regulate protein kinase C activity: A novel molecular function. Cell. Signal..

